# Hepatic SH2B1 and SH2B2 Regulate Liver Lipid Metabolism and VLDL Secretion in Mice

**DOI:** 10.1371/journal.pone.0083269

**Published:** 2013-12-17

**Authors:** Liang Sheng, Yan Liu, Lin Jiang, Zheng Chen, Yingjiang Zhou, Kae Won Cho, Liangyou Rui

**Affiliations:** Department of Molecular & Integrative Physiology, University of Michigan Medical School, Ann Arbor, Michigan, United States of America; Pennington Biomedical Research Center, United States of America

## Abstract

SH2B1 is an SH2 and PH domain-containing adaptor protein. Genetic deletion of *SH2B1* results in obesity, type 2 diabetes, and fatty liver diseases in mice. Mutations in *SH2B1* are linked to obesity in humans. SH2B1 in the brain controls energy balance and body weight at least in part by enhancing leptin sensitivity in the hypothalamus. SH2B1 in peripheral tissues also regulates glucose and lipid metabolism, presumably by enhancing insulin sensitivity in peripheral metabolically-active tissues. However, the function of SH2B1 in individual peripheral tissues is unknown. Here we generated and metabolically characterized hepatocyte-specific *SH2B1* knockout (HKO) mice. Blood glucose and plasma insulin levels, glucose tolerance, and insulin tolerance were similar between HKO, *albumin-Cre*, and *SH2B1^f/f^* mice fed either a normal chow diet or a high fat diet (HFD). Adult-onset deletion of *SH2B1* in the liver either alone or in combination with whole body *SH2B2* knockout also did not exacerbate HFD-induced insulin resistance and glucose intolerance. Adult-onset, but not embryonic, deletion of *SH2B1* in the liver attenuated HFD-induced hepatic steatosis. In agreement, adult-onset deletion of hepatic *SH2B1* decreased the expression of diacylglycerol acyltransferase-2 (DGAT2) and increased the expression of adipose triglyceride lipase (ATGL). Furthermore, deletion of liver *SH2B1* in SH2B2 null mice attenuated very low-density lipoprotein (VLDL) secretion. These data indicate that hepatic SH2B1 is not required for the maintenance of normal insulin sensitivity and glucose metabolism; however, it regulates liver triacylglycerol synthesis, lipolysis, and VLDL secretion.

## Introduction

The liver is an essential metabolic organ which produces glucose through both glycogenolysis and gluconeogenesis. During fasting, liver-produced glucose provides an essential metabolic fuel for extrahepatic tissues, including red blood cells and the brain, to meet their metabolic demands. In the fed state, insulin is released from pancreatic β cells and suppresses hepatic glucose production, thus maintaining blood glucose levels within a narrow arrange during fasting-feeding cycles. In type 2 diabetes, the ability of insulin to suppress hepatic glucose production is impaired (referred to hepatic insulin resistance), so the liver produces excessive glucose, contributing to hyperglycemia and glucose intolerance [1]. In contrast, glucagon and other counterregulatory hormones stimulate hepatic glucose production to increase blood glucose levels. Aberrant hyperglycemic responses to counterregulatory hormones may also contribute to hyperglycemia and glucose intolerance in obesity and type 2 diabetes [2].

Obesity prevalence has increased rapidly. Obesity is associated with nonalcoholic fatty liver disease (NAFLD) [3]. The liver plays a critical role in lipid metabolism. During fasting, adipose tissue releases free fatty acids which are taken up by hepatocytes and converted into ketone bodies. Ketone bodies serve as a major metabolic fuel for extrahepatic tissues in the fasted state. When carbohydrates are abundant, the liver converts glucose into fatty acids which are used to synthesize triacylglycerol (TAG). TAG is packaged into very low-density lipoprotein (VLDL) particles which deliver lipids (i.e. fatty acids and cholesterol) to extrahepatic tissues through the circulation [4]. Abnormal VLDL secretion is a risk factor for atherosclerosis [5]. Alternatively, TAG is stored in lipid droplets within hepatocytes, or is used as an intracellular energy source. Hepatic steatosis is a key risk factor for nonalcoholic steatohepatitis (NASH) [3], [6], [7]. Increased lipid content reduces hepatocyte viability and increases the expression of proinflammatory cytokines in hepatocytes [8]. Additionally, abnormal lipid accumulation impairs insulin sensitivity in the liver, contributing to increased hepatic glucose production, hyperglycemia, and glucose intolerance in obesity-associated type 2 diabetes [9].

The SH2B family contains three members of adaptor proteins (SH2B1, 2 and 3) [10]. SH2B1 and SH2B2 are ubiquitously expressed, and SH2B3 expression is restricted to the immune system [11]–[13]. SH2B1 directly binds to JAK2 and stimulates JAK2 activity, thereby enhancing JAK2 signaling in response to growth hormone, leptin, erythropoietin, and prolactin [11], [14]–[17]. SH2B1 also binds to IRS1 and IRS2 and enhances IRS protein-mediated activation of the PI 3-kinase pathway in response to leptin, insulin, and insulin-like growth factor -1 [18]–[20]. SH2B1 was also reported to mediate cell signaling in response to fibroblast growth factor, nerve growth factor, and platelet-derived growth factor [21]–[24]. Genetic disruption of the *SH2B1* gene results in severe leptin resistance, insulin resistance, obesity, type 2 diabetes, and NAFLD in mice [25], [26]. We previously reported that neuron-specific restoration of *SH2B1β* transgenes (Tg) into *SH2B1* knockout (KO) mice (called TgKO mice) fully corrects leptin resistance, hyperphagia, and obesity in TgKO mice [27]. SH2B1 directly enhances leptin signaling in cultured cells [14], [18]; therefore, neuronal SH2B1 exerts anti-obesity action at least in part by enhancing leptin sensitivity. Importantly, the metabolic function of SH2B1 is evolutionarily conserved. Deletion of *drosophila SH2B* results in obesity phenotypes in flies [28], [29]. Many *SH2B1* SNPs and missense *SH2B1* mutations have been reported to be linked to obesity and type 2 diabetes in humans [30]-[34].

We have reported that TgKO mice, which lack SH2B1 only in peripheral tissues, have normal body weight, but they are still predisposed to high fat diet (HFD)-induced insulin resistance and glucose intolerance [19], [20]. These observations raise the possibility that peripheral SH2B1 may also regulate insulin sensitivity and nutrient metabolism. In agreement, we recently reported that pancreas-specific deletion of *SH2B1* impairs HFD-induced cell expansion, thereby reducing insulin secretion capacity [35]. In this study, we examined the function of SH2B1 in the liver by generating and characterizing hepatocyte-specific *SH2B1* knockout (HKO) mice. We observed that HKO mice have relatively normal insulin sensitivity, body weight, and glucose metabolism. However, both liver lipid levels and VLDL secretion are lower in HFD-fed mice with adult-onset deletion of *SH2B1* in the liver.

## Results

### Deletion of *SH2B1* in peripheral tissues promotes hepatic steatosis

**Figure 1:**
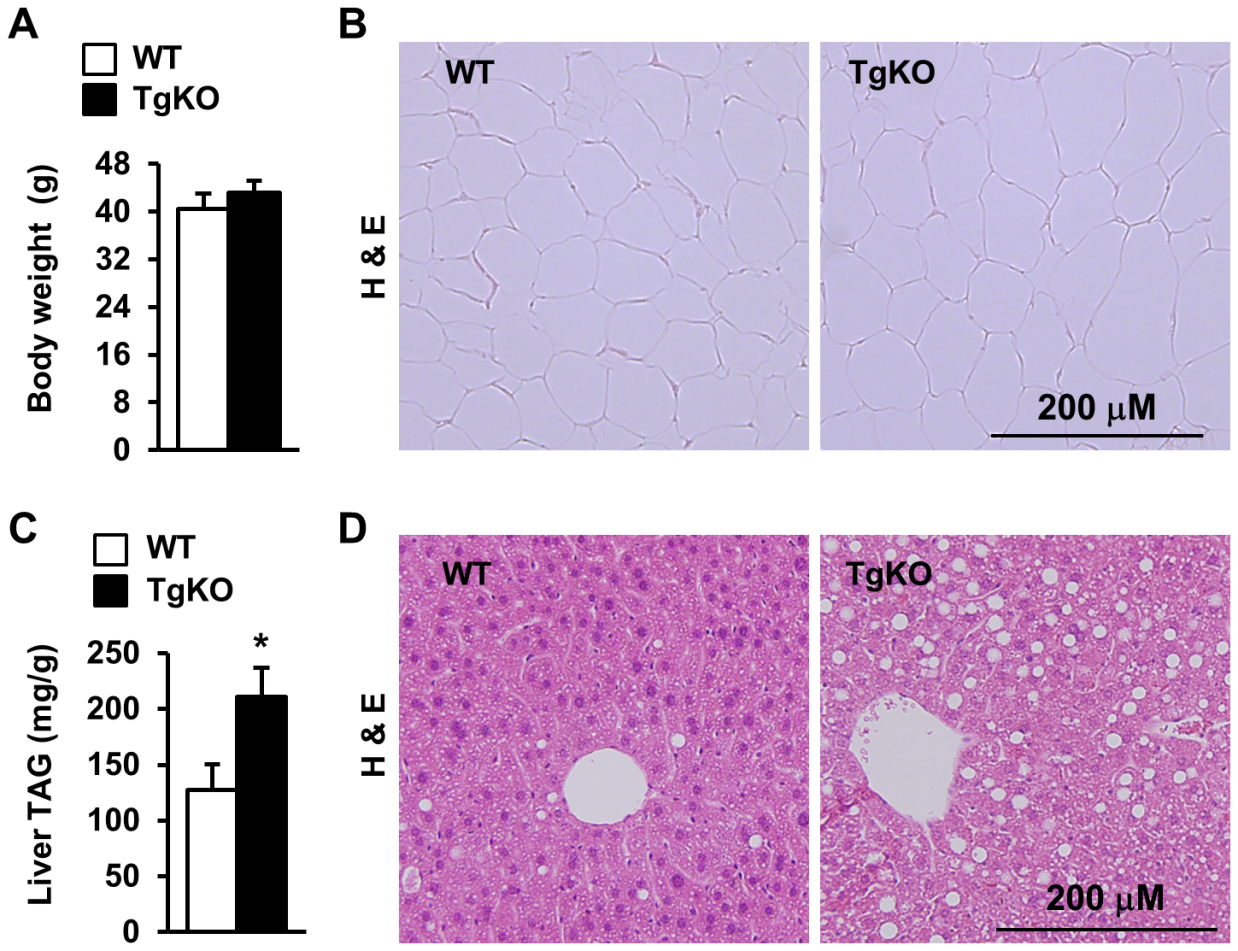
Deletion of *SH2B1* in peripheral tissues augments HFD-induced hepatic steatosis. Male mice (7 weeks of age) were fed a HFD for 16 weeks. (A) Fasting body weight. WT: n = 9, TgKO: n = 7. (B) A representative H & E staining of epididymal fat depots. (C) Liver TAG levels (normalized to liver weight). WT: n = 6; TgKO: n = 6. (D) A representative H & E staining of liver sections. Data are presented as means ± SEM. *P*<0.05.

**Figure 2:**
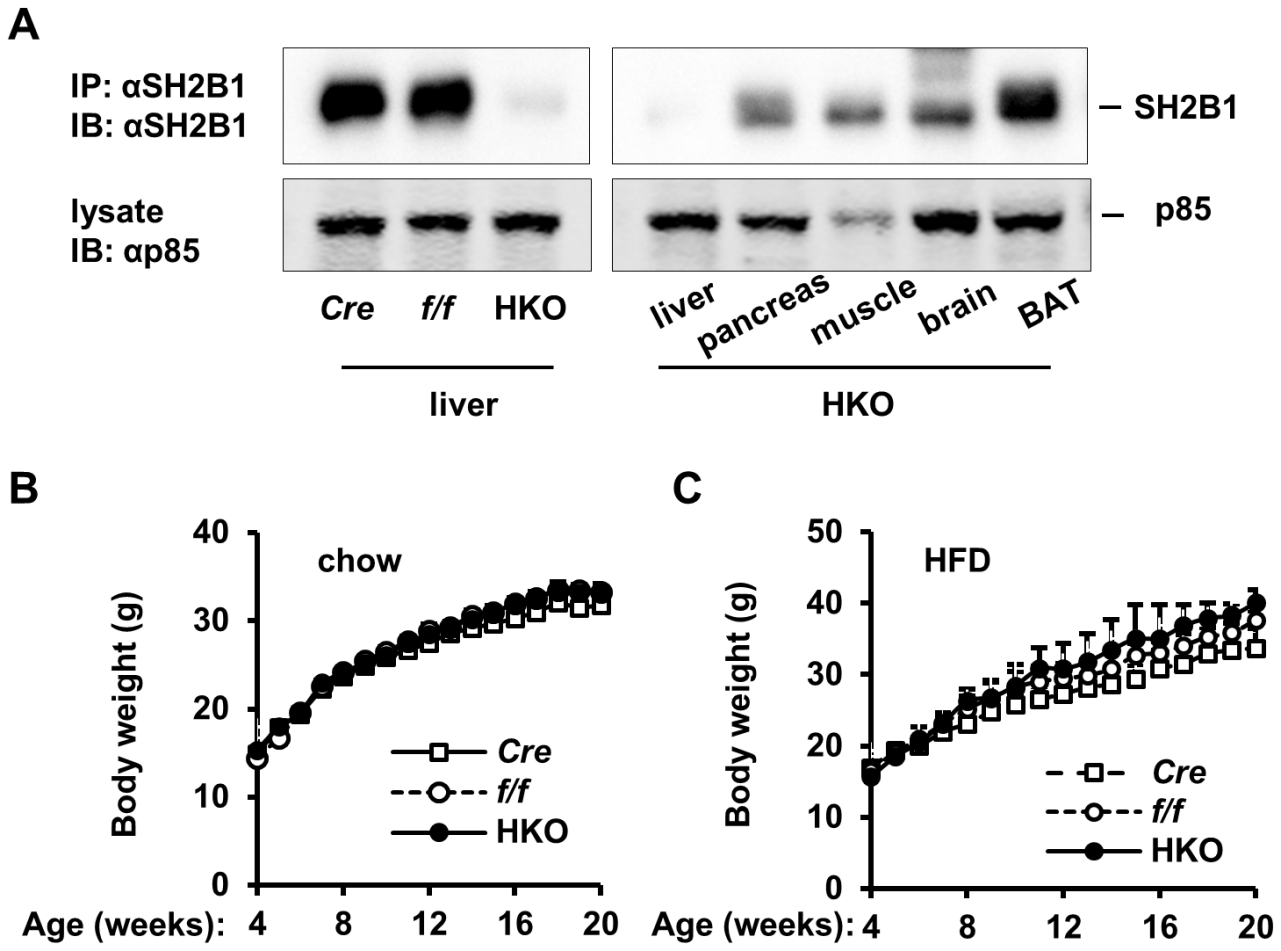
Hepatocyte-specific deletion of *SH2B1* does not increase body weight. (A) Tissue extracts were prepared from the indicated male mice (7 weeks of age) and immunoprecipitated (IP) and immunoblotted (IB) with anti-SH2B1 antibody (upper panels). Tissue extracts were also directly immunoblotted with antibody against the p85 subunit of the PI 3-kinase (lower panels). (B) Growth curves of male mice fed a normal chow diet. *f/f*: n = 9; *Cre*: n = 5; HKO: n = 9. (C) Growth curves of male mice were fed a HFD (from 7–20 weeks of age). *f/f*: n = 5; *Cre*: n = 5; HKO: n = 6. Data are presented as means ± SEM. *P*<0.05.

TgKO mice, which lack endogenous *SH2B1* but express rat *SH2B1β* transgenes under the control of the neuron-specific *enolase* promoter, were generated and verified previously [27]. SH2B1 protein was detected in the brain but not in peripheral tissues [27]. We have reported that TgKO mice are more prone to HFD-induced insulin resistance and glucose intolerance [20]. To determine whether SH2B1 deficiency in peripheral tissues promotes NAFLD, TgKO male mice (7 weeks of age) were fed a HFD for 16 weeks. Both body weight (Fig. 1A) and fat content (TgKO: 36.6%, n = 7; WT: 38.0%, n = 9; p = 0.54) were similar between TgKO and wild type (WT) mice. The sizes of individual white adipocytes were also similar between TgKO and WT mice (Fig. 2B). By contrast, liver TAG levels were 66% higher in TgKO mice than in WT mice (Fig. 1C). The livers of TgKO mice had larger lipid droplets as revealed by H & E staining of liver sections (Fig. 1D). These data suggest that peripheral SH2B1 regulates hepatic lipid metabolism independently of its action on body weight.

### Liver-specific deletion of *SH2B1* does not increase HFD-induced insulin resistance and glucose intolerance

To determine whether hepatic SH2B1 regulates glucose metabolism, we generated HKO mice using the *albumin-Cre*/loxp system. SH2B1 protein was abundant in the livers of both *albumin-Cre* and *SH2B1^f/f^* mice, but barely detectable in the livers of HKO mice (Fig. 2A). In HKO mice, SH2B1 expression was normal in the pancreas, skeletal muscle, brain, and brown adipose tissue (BAT) (Fig. 2A). HKO mice were grossly normal, and growth curves were similar between *albumin-Cre*, *SH2B1^f/f^*, and HKO mice (Fig. 2B), indicating that hepatic SH2B1 is unlikely to mediate the effect of growth hormone on promoting somatic growth. Liver-specific deletion of *SH2B1* also did not affect HFD-induced obesity in HKO mice (Fig. 2C).

**Figure 3:**
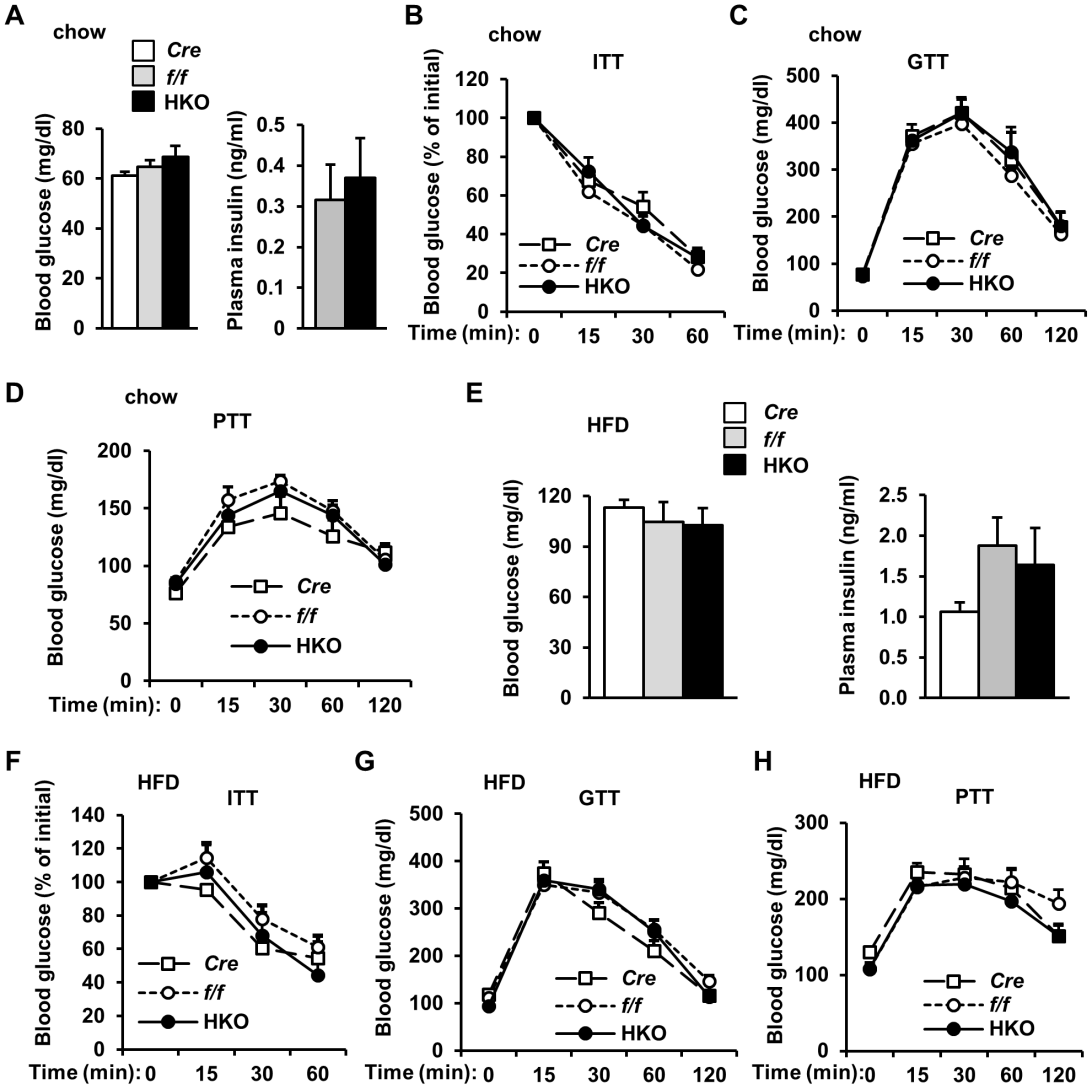
Hepatocyte-specific deletion of *SH2B1* does not exacerbate HFD-induced insulin resistance and glucose intolerance. (A-D) Mice were fed a normal chow diet. (A) Overnight fasting blood glucose and plasma insulin levels (18 weeks of age). *f/f*: n = 10; *Cre*: n = 9; HKO: n = 10. (B) ITT was performed on male mice at 18 weeks of age (insulin: 1 unit/kg body weight). *f/f*: n = 10; *Cre*: n = 7; HKO: n = 10. (C) GTT was performed on male mice at 19 weeks of age (D-glucose: 2 g/kg body weight). *f/f*: n = 10; *Cre*: n = 7; HKO: n = 10. (D) PTT was performed on mice at 19 weeks of age (pyruvate: 2 g/kg body weight). *f/f*: n = 10; *Cre*: n = 7; HKO: n = 10. (E-H) Mice (7 weeks of age) were fed a HFD. *f/f*: n = 9; *Cre*: n = 6; HKO: n = 8. (E) Overnight fasting blood glucose and plasma insulin (HFD for 11 weeks). (F) ITT (HFD for 11 weeks). (G) GTT (HFD for 12 weeks). D-glucose: 1 g/kg body weight. (H) PTT (HFD for 12 weeks). Data are presented as means ± SEM. *P*<0.05.

We metabolically characterized HKO mice using multiple approaches. Fasting blood glucose levels and plasma insulin concentrations were comparable between *albumin-Cre*, *SH2B1^f/f^*, and HKO mice (Fig. 3A). In insulin tolerance tests (ITT), exogenous insulin similarly decreased blood glucose in *albumin-Cre*, *SH2B1^f/f^*, and HKO mice (Fig. 3B). In glucose tolerance tests (GTT), glucose injection increased blood glucose to comparable levels among these three genotypes (Fig. 3C). To estimate hepatic glucose production, we performed pyruvate tolerance tests (PTT). Administration of pyruvate, a gluconeogenic precursor, increased blood glucose to a similar extent between these three groups (Fig. 3D).

To determine whether hepatic SH2B1 is involved in HFD-induced insulin resistance, we fed HKO and control male mice a HFD. Overnight fasting blood glucose and plasma insulin levels were similar between *albumin-Cre*, *SH2B1^f/f^*, and HKO mice (Fig. 3E). Insulin tolerance (Fig. 3F), glucose tolerance (Fig. 3G), and pyruvate tolerance (Fig. 3H) were also indistinguishable between these three genotypes.

**Figure 4:**
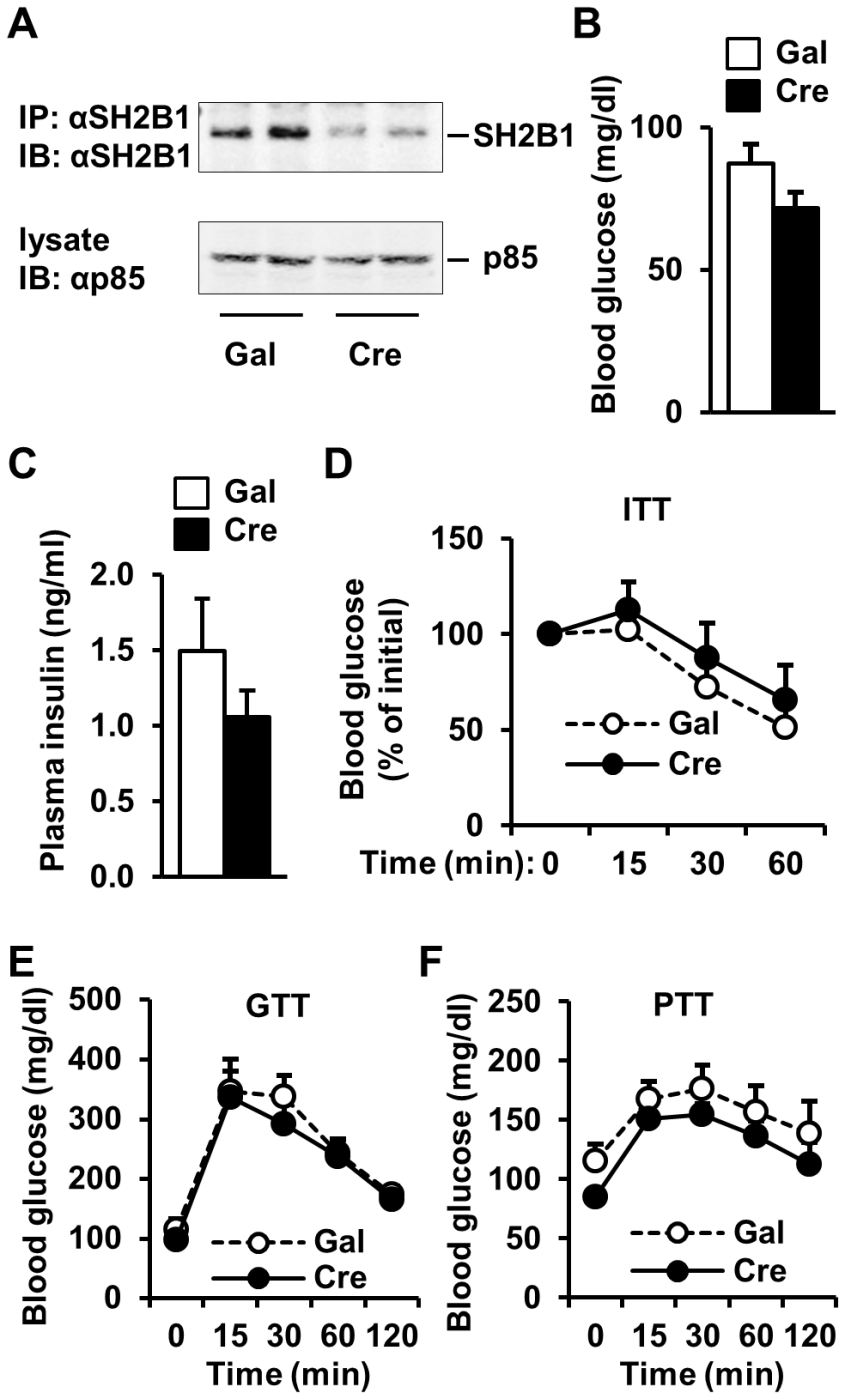
Adult-onset deletion of liver *SH2B1* does not exacerbate HFD-induced insulin resistance and glucose intolerance. (A) Tissue extracts were prepared from male mice (7 weeks of age) 7 days after adenoviral infection. Tissue extracts were immunoprecipitated (IP) and immunoblotted (IB) with anti-SH2B1 antibody (upper panel). Tissue extracts were also immunoblotted with anti-p85 antibody (lower panel). (B-E) *SH2B1^f/f^* male mice (7 weeks of age) were fed a HFD for 5 weeks and infected with β-gal (n = 7) or Cre adenoviruses (n = 6) via tail vein injection. (B-C) Overnight fasting blood glucose (B) and plasma insulin levels (C) 10 days after adenoviral infection. (D) ITT (13 days after adenoviral infection). (E) GTT (16 days after adenoviral infection). (F) PTT (19 days after adenoviral infection).

Embryonic onset deletion of hepatic *SH2B1* may cause a developmental adaptation in HKO mice. To address this concern, we deleted *SH2B1* in the livers of adult *SH2B1^f/f^* mice using Cre adenoviral infection. *SH2B1^f/f^* male mice (7 weeks of age) were fed a HFD for 5 weeks, and infected with Cre or β-glactosidase (Gal) adenoviruses via tail vein injection. Cre but not Gal adenoviral infection markedly reduced SH2B1 protein levels in the liver (Fig. 4A). Fasting blood glucose and plasma insulin levels were similar between Cre and Gal groups 10 days after infection (Figs. 4B, C). Insulin, glucose, and pyruvate tolerance tests were also indistinguishable between Cre and Gal adenovirus-infected mice (Figs. 4D-F). Taken together, these data indicate that hepatic SH2B1 is dispensable for insulin regulation of glucose metabolism in mice under either normal or obese conditions.

### Deletion of both *SH2B1* and *SH2B2* in the liver does not increase HFD-induced insulin resistance and glucose intolerance

**Figure 5:**
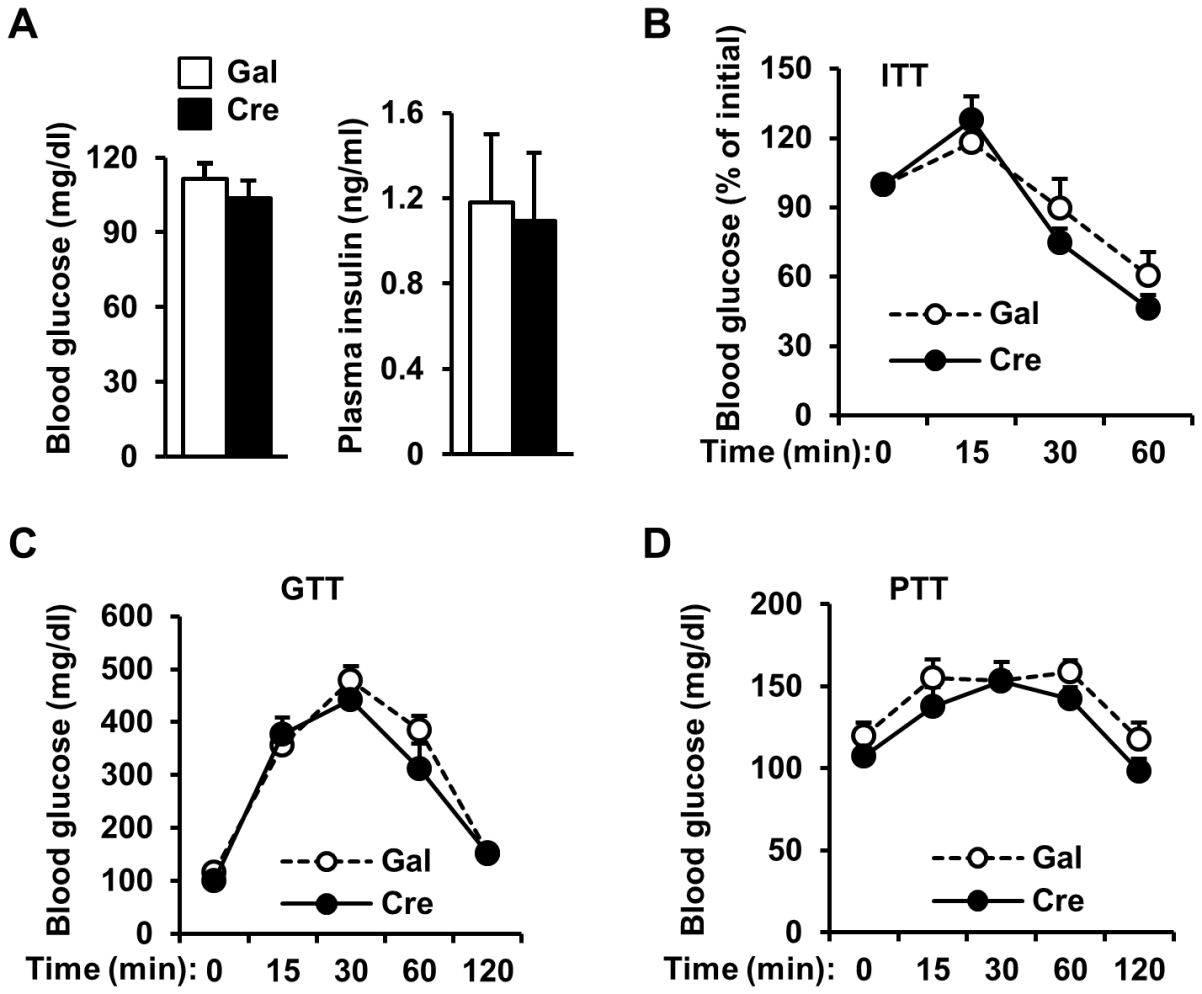
Deletion of both *SH2B1* and *SH2B2* in the liver does not exacerbate HFD-induced insulin resistance and glucose intolerance. *SH2B1^f/f^:SH2B2^KO^* male mice (7 weeks of age) were fed a HFD for 5 weeks and infected with β-gal (n = 6) or Cre adenoviruses (n = 6) via tail vein injection. (A) Overnight fasting blood glucose and plasma insulin levels 10 days after adenoviral infection. (B) ITT (13 days after adenoviral infection). (C) GTT (16 days after adenoviral infection). D-glucose: 1 g/kg body weight. (D) PTT (19 days after adenoviral infection). Data are presented as means ± SEM. *P*<0.05.

In HKO mice, SH2B2 (also called APS) may have a similar metabolic function and compensate for SH2B1 deficiency. To address this possibility, we deleted hepatic *SH2B1* in *SH2B2* knockout (*SH2B2^KO^*) mice. *SH2B2^KO^* mice were generated previously and reported to have slightly enhanced insulin sensitivity [12], [36]. *SH2B2^KO^* mice were crossed with *SH2B1^f/f^* to generate *SH2B1^f/f^:SH2B2^KO^* double mutant mice. *SH2B1^f/f^:SH2B2^KO^* male mice (7 weeks of age) were fed a HFD for 5 weeks, and infected with Cre or Gal adenoviruses via tail vein injection. Overnight fasting blood glucose and plasma insulin levels were similar between Cre and Gal groups 10 days after infection (Fig. 5A). Responses to insulin (ITT), glucose (GTT), and pyruvate injection (PTT) were also indistinguishable between Cre and Gal adenovirus-infected mice (Figs. 5B, C, D). These data indicate that SH2B1 and SH2B2 in hepatocytes are not required for insulin suppression of hepatic glucose production and systemic glucose homeostasis even under HFD conditions.

### Hepatocyte-specific deletion of *SH2B1* does not protect against dietary liver injury

**Figure 6:**
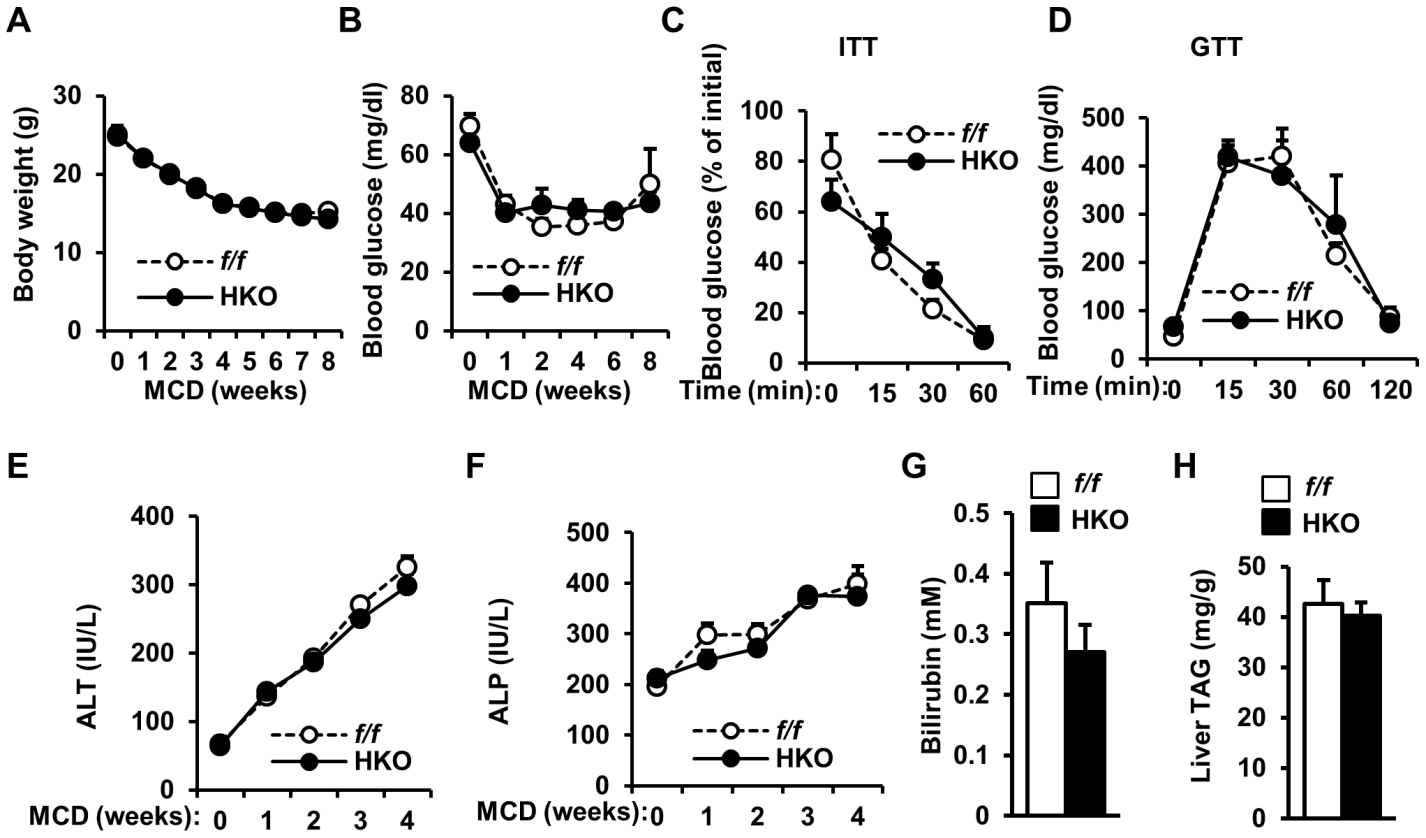
Liver-specific deletion of *SH2B1* does not augment MCD-induced liver injury. Male mice (7 weeks of age) were fed a MCD. (A) Growth curves. *f/f*: n = 5; HKO: n = 7. (B) Overnight fasting blood glucose. *f/f*: n = 5; HKO: n = 5. (C) ITT (MCD for 7 weeks). *f/f*: n = 5; HKO: n = 5. (D) GTT (MCD for 6 weeks). *f/f*: n = 5; HKO: n = 5. (E) Plasma ALT activity. *f/f*: n = 5; HKO: n = 5. (F). Plasma ALP activity. *f/f*: n = 5; HKO: n = 5. (G) Plasma bilirubin levels (MCD for 8 weeks). *f/f*: n = 5; HKO: n = 5. (H) Liver TAG levels (MCD for 8 weeks). *f/f*: n = 5; HKO: n = 5. Data are presented as means ± SEM. *P*<0.05.

In cultured cells, SH2B1 promotes both the JAK/STAT and the PI 3-kinase/Akt pathways [14], [15], [18], [20]. These two pathways play a critical role in protecting against hepatocyte injury and promoting liver regeneration [37], [38]. To determine whether hepatic SH2B1 protects against liver injury, we fed HKO male mice (7 weeks of age) a methionine- and choline-deficient diet (MCD). MCD is commonly used to induce liver injury and NASH. Both MCD-fed HKO and *SH2B1^f/f^* mice similarly lost their body weights (Fig. 6A). MCD feeding reduced fasting blood glucose levels to a similar degree between *SH2B1^f/f^* and HKO mice (Fig. 6B). Responses to insulin (ITT) and glucose (GTT) injection were also indistinguishable between *SH2B1^f/f^* and HKO mice (Figs. 6C, D). Blood alanine aminotransferase (ALT) and alkaline phosphatase (ALP) activities are commonly used as biomarkers to estimate liver injury. MCD feeding similarly increased the activities of blood ALT and ALP in both *SH2B1^f/f^* and HKO mice (Figs. 6E, F). Blood bilirubin levels and liver TAG content were also similar between *SH2B1^f/f^* and HKO mice (Figs. 6G, H). These data indicate that hepatic SH2B1 is not required for the maintenance of liver integrity and protection against liver injury.

### Adult-onset, acute deletion of *SH2B1* in the liver attenuates HFD-induced hepatic steatosis

**Figure 7:**
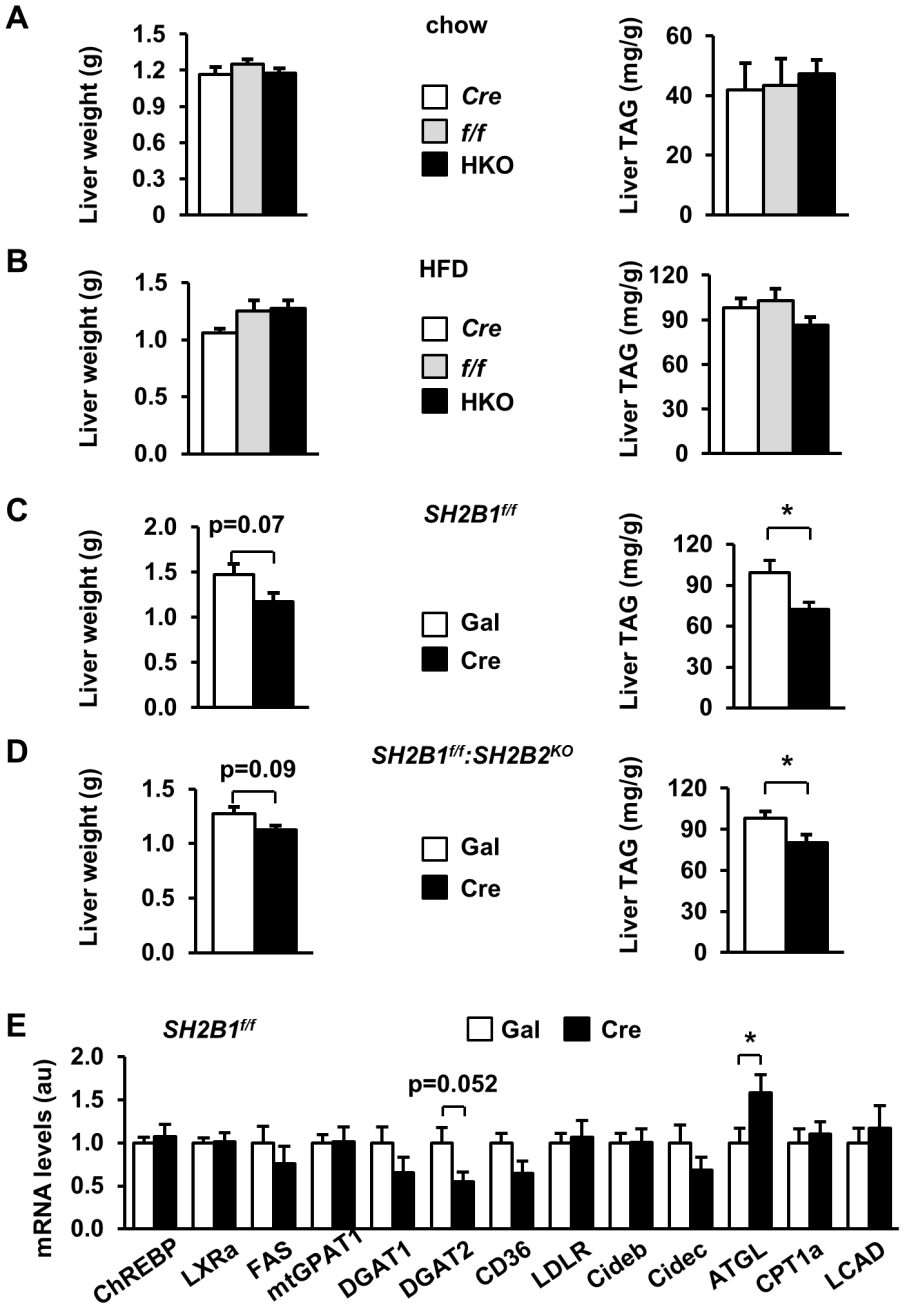
Hepatocyte-specific deletion of *SH2B1* attenuates HFD-induced hepatic steatosis. (A) Liver weight and liver TAG levels (normalized to liver weight) of overnight-fasted male mice (21 weeks of age). *f/f*: n = 10; *Cre*: n = 7; HKO: n = 10. (B) Male mice (7 weeks of age) were fed a HFD for 14 weeks. Liver weight and TAG levels of overnight-fasted mice. *f/f*: n = 10; *Cre*: n = 7; HKO: n = 10. (C) *SH2B1^f/f^* male mice (7 weeks of age) were fed a HFD for 5 weeks and infected with β-gal (n = 7) or Cre adenoviruses (n = 6) via tail vein injection. Liver weight and TAG levels were measured in fasted mice 29 days after adenoviral infection. (D) *SH2B1^f/f^:SH2B2^KO^* male mice (7 weeks of age) were fed a HFD for 5 weeks and infected with β-gal (n = 6) or Cre adenoviruses (n = 6). Liver weight and TAG levels were measured in fasted mice 29 days after adenoviral infection. (E) Total liver mRNA was prepared from the mice described in (C) and used to measure mRNA levels of the indicated genes by qPCR and normalized to *β-actin* expression. The values were further normalized to the β-gal group. Data are presented as means ± SEM. *P*<0.05.

To examine the role of hepatic SH2B1 in liver lipid metabolism, we measured liver weight and liver TAG levels. Both liver weight and TAG content were similar between *albumin-Cre*, *SH2B1^f/f^*, and HKO mice fed either a normal chow diet (Fig. 7A) or a HFD for 14 weeks (Fig. 7B).

Embryonic deletion of hepatic *SH2B1* in HKO mice may cause a developmental adaptation to compensate for SH2B1 deficiency. Therefore, we measured liver weight and TAG levels in mice with adult-onset deletion of liver *SH2B1*. *SH2B1^f/f^* male mice were fed a HFD for 5 weeks and then infected with Cre adenoviruses to delete *SH2B1* in the liver as described in Fig. 4. Liver weight was lower in the Cre group than in the Gal group (p = 0.07) 29 days after adenoviral infection; accordingly, liver TAG levels were significantly lower in mice with adult-onset deletion of liver *SH2B1* (Fig. 7C). In a separate mouse model, adult *SH2B1^f/f^:SH2B2^KO^* male mice were fed a HFD for 5 weeks and infected with Cre or Gal adenoviruses. Adult-onset deletion of liver *SH2B1* similarly attenuated HFD-induced hepatic steatosis in *SH2B2* KO mice 29 days after infection (Fig. 7D). Together, these data indicate that hepatic SH2B1 promotes hepatic lipid accumulation in adult mice fed a HFD, which may contribute to obesity-associated NAFLD.

To gain insights into the potential mechanisms of SH2B1 action, we measured the expression of key genes that regulate lipid metabolism in the liver. The expression of most lipogenic genes (e.g. *ChREBP*, *LXRa*, *FAS*, *mtGPAT1*, and *DGAT1*) as well as the genes that regulate lipid uptake (e.g. *CD36* and *LDLR*), lipid droplet activity (*Cideb* and *Cidec*), and fatty acid β oxidation (e.g *CPT1a* and *LCAD*) was similar between Cre and Gal adenovirus-infected *SH2B1^f/f^* mice (Fig. 7E). However, the expression of diacylglycerol acyltransferase-2 (DGAT2), an enzyme which synthesizes TAG, was lower in Cre adenovirus-infected *SH2B1^f/f^* mice (Fig. 7E). In contrast, the expression of adipose triglyceride lipase (ATGL), a key lipolytic enzyme, was significantly higher in Cre adenovirus-infected *SH2B1^f/f^* mice (Fig. 7E). These data suggest that deletion of hepatic *SH2B1* may decrease TAG synthesis and increase lipolysis, leading to lower hepatic lipid content in mice with adult-onset deletion of liver *SH2B1*.

### Deletion of both *SH2B1* and *SH2B2* in the liver attenuates VLDL secretion

**Figure 8:**
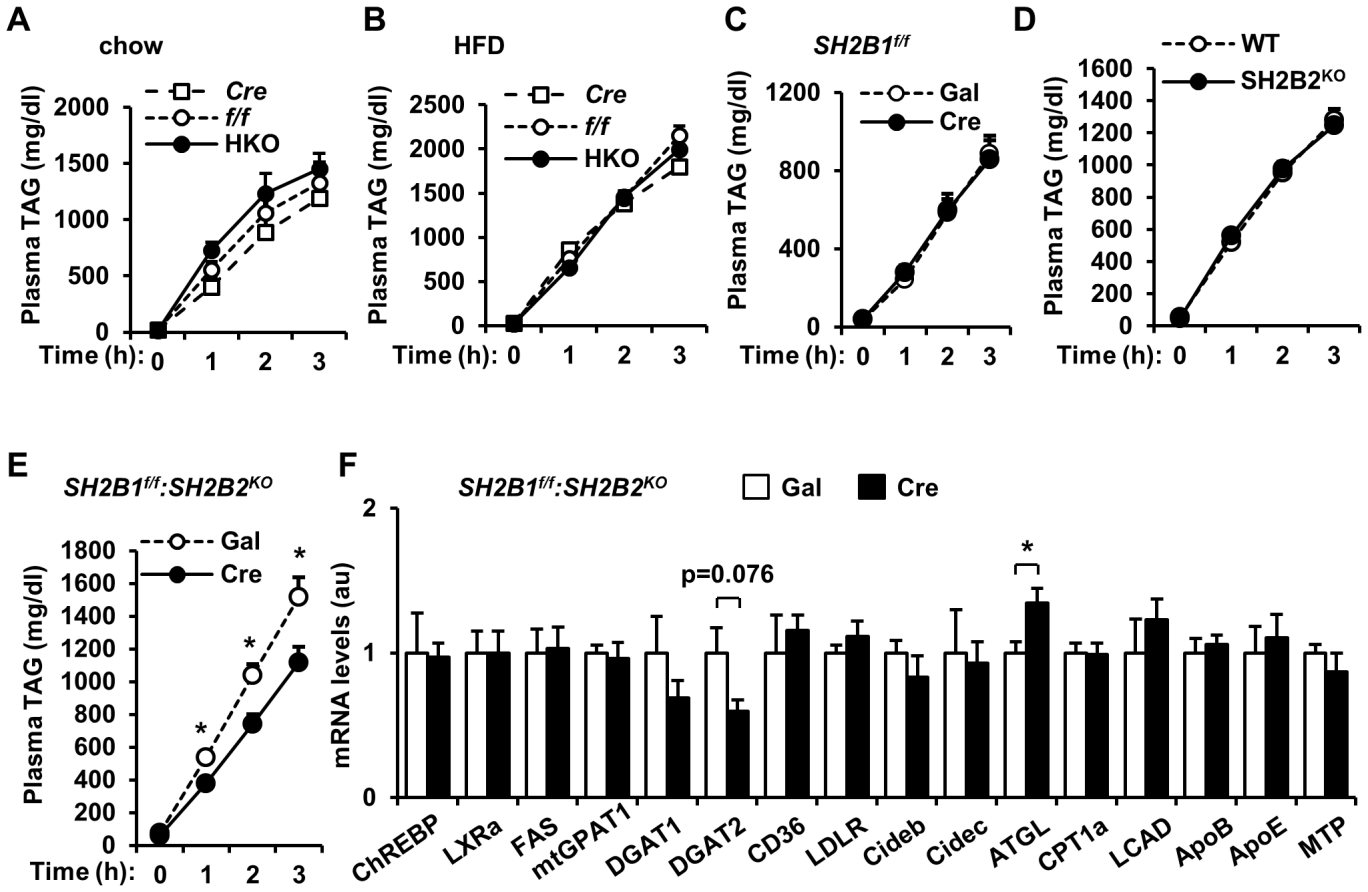
Deletion of both *SH2B1* and *SH2B2* in the liver suppresses VLDL secretion. (A) VLDL secretion in the mice (20 weeks of age) described in Fig. 7A. (B) Male mice (7 weeks of age) were fed a HFD for 13 weeks as described in Fig. 7B, and VLDL secretion was measured. (C) *SH2B1^f/f^* male mice were fed a HFD and infected with β-gal (n = 7) or Cre adenoviruses (n = 6) as described in Fig. 7C. VLDL secretion was measured 22 days after adenoviral infection. (D) VLDL secretion was measured in *SH2B2* knockout and WT males (11 weeks of ages). SH2B2^KO^: n = 5; WT: n = 5. (E-F) *SH2B1^f/f^:SH2B2^KO^* male mice were fed a HFD and infected with β-gal (n = 6) or Cre adenoviruses (n = 6) as described in Fig. 7D. (E) VLDL secretion was measured 22 days after adenoviral infection. (F) The expression of the indicated genes was measured by qPCR and normalized to *β-actin* expression. Data are presented as means ± SEM. *P*<0.05.

High TAG levels promote VLDL secretion from hepatocytes [39]. To examine the role of hepatic SH2B1 in VLDL secretion, HKO, *albumin-Cre*, and *SH2B1^f/f^* male mice were fed either a chow diet or a HFD for 14 weeks. Mice were treated with Triton WR1339 to block VLDL clearance, and VLDL secretion was estimated by measuring blood TAG levels after Triton WR1339 treatment. VLDL secretion was similar between HKO, *albumin-Cre*, and *SH2B1^f/f^* male mice fed either a normal chow (Fig. 8A) or a HFD (Fig. 8B). Even though adult-onset deletion of liver *SH2B1* attenuated HFD-induced hepatic steatosis in obese *SH2B1^f/f^* mice as described in Fig. 7C, it did not decrease VLDL secretion (Fig. 8C). Systemic deletion of *SH2B2* also did not decrease VLDL secretion in *SH2B2^KO^* mice fed a HFD (Fig. 8D). To determine whether deletion of both *SH2B1* and *SH2B2* in the liver affect VLDL secretion, *SH2B1^f/f^:SH2B2^KO^* male mice were fed a HFD for 5 weeks and infected with Cre or Gal adenoviruses as described in Fig. 7D. Simultaneous deletion of both *SH2B1* in the liver and *SH2B2* in the whole body significantly reduced VLDL secretion (Fig. 8E). These data suggest that SH2B1 and SH2B2 in the liver may have a redundant function in promoting VLDL secretion; therefore, deletion of both, but not *SH2B1* or *SH2B2* alone, impairs VLDL secretion.

To gain insights into the potential mechanisms of SH2B1 regulation of VLDL secretion, we measured the expression of key genes which regulate lipid metabolism and VLDL secretion in the livers of Cre or Gal adenovirus-infected *SH2B1^f/f^:SH2B2^KO^* mice. In agreement with Fig. 7E, the expression of *ChREBP*, *LXRa*, *FAS*, *mtGPAT1*, *DGAT1*, *CD36*, *LDLR*, *Cideb*, *Cidec*, *CPT1a*, *LCAD*, *ApoB*, *ApoE*, and *MTP* was similar between Cre and Gal groups; DGAT2 expression was lower, whereas ATGL expression was higher, in the Cre group (Fig. 8F). Surprisingly, the expression of *ApoB*, *ApoE*, and *MTP*, which promote VLDL secretion, was similar between Cre and Gal groups (Fig. 8F).

## Discussion

SH2B1, a ubiquitously expressed adaptor protein, is a key metabolic regulator, and its metabolic function is conserved from insects to humans [25], [28], [33]. Impaired SH2B1 function is associated with obesity and type 2 diabetes in humans [30]–[34]. In this study, we have elucidated a new function of SH2B1 in the liver.

SH2B1 was highly expressed in the liver. It binds to both insulin receptors and IRS proteins, and enhances IRS protein-mediated activation of the PI 3-kinase pathway in cultured cells [18]–[20], [40], [41]. We hypothesized that hepatic SH2B1 increases the ability of insulin to suppress hepatic glucose production. Surprisingly, hepatocyte-specific deletion of *SH2B1* did not cause hyperinsulinemia, hyperglycemia, insulin resistance, or glucose intolerance in HKO mice. Unlike TgKO mice which are prone to HFD-induced insulin resistance, HFD-fed HKO mice developed insulin resistance, glucose intolerance, and pyruvate intolerance to a similar degree as *albumin-Cre* and *SH2B1^f/f^* mice fed a HFD. Adult-onset deletion of hepatic *SH2B1* also did not exacerbate HFD-induced insulin resistance and glucose intolerance in mice. Furthermore, simultaneous deletion of both hepatic *SH2B1* and whole body *SH2B2* neither caused insulin resistance nor exacerbated HFD-induced insulin resistance and glucose intolerance. These data indicate that hepatic SH2B1 is dispensable for insulin regulation of glucose metabolism in either normal or obese mice. Since deletion of *SH2B1* in the entire peripheral tissues exacerbates HFD-induced insulin resistance and glucose intolerance in TgKO mice [20], SH2B1 in other peripheral tissues (e.g. skeletal muscle and/or adipose tissue) is likely to promote insulin regulation of glucose metabolism.

SH2B1 directly enhances growth hormone signaling and promotes activation of both the JAK2/STAT and the PI 3-kinase pathways in cultured cells [11], [18], [42]. The JAK2/STAT and the PI 3-kinase pathways regulate liver injury and regeneration [37], [38]. Thus, we hypothesized that hepatic SH2B1 may protect against liver injury as well as mediate growth hormone stimulation of somatic growth. However, HKO mice had normal growth rates and body weight. Hepatocyte-specific deletion of *SH2B1* also did not exacerbate MCD-induced liver injury in HKO mice. These results indicate that hepatic SH2B1 is not required for liver homeostasis and somatic growth.

We also hypothesized that hepatic SH2B1 directly promotes lipogenesis in hepatocytes by enhancing insulin action. However, liver size and liver TAG levels were similar between HKO, *albumin-Cre*, and *SH2B1^f/f^* mice fed either a normal chow or a HFD. Unlike embryonic deletion of hepatic *SH2B1* in HKO mice, adult-onset deletion of hepatic *SH2B1* attenuated HFD-induced hepatic steatosis in both *SH2B1^f/f^* and *SH2B1^f/f^:SH2B2^KO^* mice. It is likely that embryonic deletion of hepatic *SH2B1* causes a developmental adaptation which compensates for SH2B1 deficiency in the livers of HKO mice. Adult-onset deletion of hepatic *SH2B1* did not decrease the expression of most insulin-regulated lipogenic genes, but it decreased DGAT2 expression and increased ATGL expression. These observations raise the possibility that hepatic SH2B1 stimulates DGAT2-mediated TAG synthesis and inhibits ATGL-mediated lipolysis, thus increasing hepatic lipid content and/or VLDL secretion.

Interestingly, deletion of *SH2B1* in the entire peripheral tissues augmented HFD-induced hepatic steatosis in TgKO mice. Hepatic steatosis in TgKO mice may be explained by SH2B1 regulation of crosstalk between different metabolic tissues. For instance, adipose SH2B1, which is present in HKO but not in TgKO mice, may regulate adipose lipolysis and/or endocrine function, thus indirectly regulating lipid metabolism in the liver. Unlike HKO mice, TgKO mice developed more severe insulin resistance and hyperinsulinemia than WT control mice upon feeding a HFD. Hyperinsulinemia may counteract hepatic SH2B1-deficiency and stimulate lipogenesis and hepatic steatosis in TgKO mice.

Deletion of either *SH2B1* in the liver or *SH2B2* in whole body alone did not decrease VLDL secretion; however, simultaneous deletion of both significantly reduced VLDL secretion in *SH2B1^f/f^:SH2B2^KO^* mice. Therefore, hepatic SH2B1 and SH2B2 are likely to act redundantly to promote VLDL production and/or secretion. Lower liver TAG levels in the mutant mice are likely to contribute to reduced VLDL secretion. However, deletion of *SH2B1* alone in *SH2B1^f/f^* mice decreased only hepatic TAG levels but not VLDL secretion, suggesting that hepatic SH2B1 and SH2B2 promote VLDL secretion by additional mechanisms. Insulin signaling is unlikely to mediate SH2B1/SH2B2-promotion of VLDL secretion, because insulin suppresses VLDL secretion. It will be interesting to determine whether JAK2 mediates SH2B1 and SH2B2 regulation of VLDL secretion in the future.

In summary, we report that liver-specific deletion of *SH2B1* does not affect hepatic glucose production, systemic insulin sensitivity, and glucose intolerance. Adult onset, but not embryonic, deletion of hepatic *SH2B1* attenuates HFD-induced hepatic steatosis. Deletion of both, but not hepatic *SH2B1* or *SH2B2* alone, impairs VLDL secretion. Our data indicate that hepatic SH2B1 is a novel regulator of TAG biosynthesis, lipolysis, and VLDL secretion.

## Materials and Methods

### Mouse Models

Animal experiments were conducted following the animal protocols approved by the University Committee on Use and Care of Animals (UCUCA) at the University of Michigan. TgKO mice were generated and verified previously [27]. Hepatocyte-specific *SH2B1* knockout (HKO) mice were generated using the Cre/loxP system. *SH2B1^f/f^* mice have been generated and verified previously [35]. One loxP site was inserted into the intron between the second and third exons of *SH2B1* and a second loxP site was inserted into the intron between the fifth and sixth exons. Exons 2–5 encode amino acids 1–436 of all four SH2B1 isoforms. HKO mice (genotype: *SH2B1^f/f^;Cre^+/−^*) were generated by crossing *SH2B1^f/f^* with *albumin-Cre* mice (C57BL/6 genetic background). Mice were housed on a 12-hour light/12-hour dark cycle in the Unit for Laboratory Animal Medicine at the University of Michigan, and fed either a standard rodent chow diet (9% fat; Lab Diet, St. Louis, MO), HFD (60% fat, D12492, Research Diets, New Brunswick, NJ) or MCD (A02082002B, New Brunswick, NJ) *ad libitum* with free access to water.

### Animal experiments

Tail blood samples were collected. Plasma insulin levels were measured using a rat insulin ELISA kit (Crystal Chem Inc., Downers Grove, IL). Plasma ALT, ALP, and bilirubin levels were measured using an ALT reagent set, an ALP reagent set, and a total bilirubin reagent set (Pointe Scientific Inc., Canton, MI), respectively. Blood TAG levels were measured using a TAG assay kit (Pointe Scientific Inc., Canton, MI). Glucose tolerance tests (GTT), insulin tolerance tests (ITT), and pyruvate tolerance tests were conducted as previously described [2]. Mice were anesthetized with avertin (2.5 g tribromoethanol and 5 ml *tert*-amyl alcohol in 200 ml of water; 0.02 ml/g of body weight) and euthanized, and tissues were harvested and stored at −80°C.

### H & E staining

Paraffin sections of liver and adipose tissue were stained with hematoxylin and eosin (H & E) and visualized using a BX51 microscope equipped with a DP70 Digital Camera (Olympus, Tokyo, Japan).

### TAG concentrations

Liver samples were homogenized in 1% acetic acid. Cell lysates were extracted by chloroform:methanol (2∶1). The organic phase was transferred to a new tube and dried by evaporation. Lipid residues were dissolved in isopropanol and measured using a TAG assay kit (Pointe Scientific Inc., Canton, MI).

### VLDL secretion assays

Mice were fasted for 5 h and then intravenously injected with Triton WR1339 (Sigma-Aldrich, St. Louis, MO) at 600 mg/kg body weight. Blood samples were collected 0–3 h after injection and used to measure TAG concentrations.

### Immunoprecipitation and immunoblotting

Frozen tissue samples were homogenized in ice-cold lysis buffer (50 mM Tris HCl, pH 7.5, 0.5% Nonidet P-40, 150 mM NaCl, 2 mM EGTA, 1 mM Na_3_VO_4_, 100 mM NaF, 10 mM Na_4_P_2_O_7_, 1 mM phenylmethylsulfonyl fluoride, 10 μg/ml aprotinin, 10 μg/ml leupeptin). Tissue extracts were immunoprecipitated and immunoblotted with the indicated antibodies. Antibody dilution ratios for immunoblotting were: SH2B1 (Laboratory-generated): 1∶10,000; p80 (Laboratory-generated): 1∶10,000.

#### Quantitative Real-time RT-PCR (qPCR)

qPCR was performed using absolute QPCR SYBR Green kits (Thermo Scientific, Waltham, MA) and Mx3000P real-time PCR system (Stratagene, La Jolla, CA) as described previously [2]. Primer sequences are: CPT-1a forward: 5′-CTGATGACGGCTATGGTGTTT-3′, reverse: 5′-GTGAGGCCAAACAAGGTGATA-3′; FAS forward: 5′-TTGACGGCTCACACACCTAC-3′, reverse: 5′-CGATCTTCCAGGCTCTTCAG-3′; ChREBP forward: 5′-CTGGGGACCTAAACAGGAGC-3′, reverse: 5′-GAAGCCACCCTATAGCTCCC-3′; LXRa forward: 5′-GAGTTCTCCAGAGCCATGAATG-3′, reverse: 5′-ATATGTGTGTTGCAGCCTCTCT-3′; mtGPAT1 forward: 5′-ACGCTGAGAGTGCCACATACT-3′, reverse: 5′-GAGAGATCGCTACAGCACCAC-3′; DGAT1 forward: 5′-CGTGGTATCCTGAATTGGTG-3′, reverse: 5′-GGCGCTTCTCAATCTGAAAT-3′; DGAT2 forward: 5′-ATCTTCTCTGTCACCTGGCT-3′, reverse: 5′-ACCTTTCTTGGGCGTGTTCC-3′; CD36 forward: 5′-GGAGTGGTGATGTTTGTTGCT-3′, reverse: 5′-GCACACACCACCATTTCTTCT-3′; LDLR forward: 5′-CAGAAGACCACAGAGGACGAG-3′, reverse: 5′-GGGAGGTCTGGAGAGAGTGTC-3′; Cideb forward: 5′-GACCCTTCCGTGTCTGTGAT-3′, reverse: 5′-GTAGCAGCAAGGTCTCCAGG-3′; Cidec forward: 5′-CCTATGACCTGCACTGCTACAAG-3′, reverse: 5′-CATGTAGCTGGAGGTGCCAAG-3′; ATGL forward: 5′-TTCACCATCCGCTTGTTGGAG-3′, reverse: 5′-AGATGGTCACCCAATTTCCTC-3′; ApoB forward: 5′-CCAGAGTGTGGAGCTGAATGT-3′, reverse: 5′-TTGCTTTTTAGGGAGCCTAGC-3′; ApoE forward: 5′-CTGAACCGCTTCTGGGATTAC-3′, reverse: 5′-TCCGTCATAGTGTCCTCCATC-3′; MTP forward: 5’-CTCCACAGTGCAGTTCTCACA-3′, reverse: 5′- AGAGACATATCCCCTGCCTGT-3′; LCAD forward: 5′-CACTCAGATATTGTCATGCCCT-3′, reverse: 5′-TCCATTGAGAATCCAATCACTC-3′; β-actin forward: 5′-AAATCGTGCGTGACATCAAA-3′, reverse: 5′-AAGGAAGGCTGGAAAAGAGC-3′.

### Statistical Analysis

Data are presented as means ± SEM. Differences between groups were determined by two-tailed Student's *t* tests. *P*<0.05 was considered significant.
